# Prevalence and Immunization Status of Hepatitis B Virus in the HIV Cohort in Fife, Scotland

**DOI:** 10.4021/jocmr2009.12.1282

**Published:** 2010-02-08

**Authors:** Lukman Hakeem, Grace Thomson, Eleanor McCleary, Diptendu Bhattacharyya, Indranil Banerjee

**Affiliations:** a Infectious Diseases Unit, Victoria Hospital (NHS Fife), Kirkcaldy, Fife, Scotland, UK; b Genitourinary Medicine, The Beeches Centre, Forth Park Hospital, Kirkcaldy, Fife, Scotland, UK

## Abstract

**Background:**

Routes of transmission of hepatitis B virus (HBV)/HIV infections are similar and there is a significant rate of co-infection in patients. A study was recently carried out in NHS Fife, Scotland from February 2007 - February 2008 to estimate the prevalence of HBV/HIV co-infection, occult HBV infection and immunisation status against HBV in a cohort of patients with HIV attending the departments of infectious diseases and genitourinary medicine.

**Methods:**

Case notes were reviewed retrospectively (n = 70). Details on patient demographics, risk category, nadir/current CD4 count, HIV viral load and vaccination history were analysed. HBV markers (HBsAg/anti-HBs/anti-HBc/HBV DNA) and alanine transaminase (ALT) levels were tested prospectively if these tests had not been carried out in the previous 12 months.

**Results and Conclusion:**

Prevalence of HBV/HIV co-infection was 5.6% of which 2.8% of patients had occult infection and 22.9% had evidence of previous exposure. Although HBV is preventable by vaccination, only 24.2% of patients had been vaccinated against it. Improvements could therefore be made in the field of prevention with vaccination and monitoring the immune response in this cohort.

**Keywords:**

Prevalence; Immunization status; Hepatitis B Virus; HIV

## Introduction

There are estimated 350 million hepatitis B virus (HBV) carriers and around 40 million HIV infected patients worldwide [1,2]. Routes of transmission for these two infections are similar and there is a significant rate of co-infection in patients. Underlying HIV infection decreases the rate of HBV clearance, and increases the chance of HBV chronicity and progression [3,4]. British HIV Association (BHIVA) guidelines therefore recommend that all HIV infected individuals should be tested for HBV serum markers, in part to determine which individuals should receive vaccination against this virus [5].

There is no comprehensive data from the UK defining HIV/HBV co-infection rates. A study in 631 HIV positive people, most of whom were homosexual men, in London showed a dual HIV/HBV infection rate of 6% [6]. In many parts of Africa, HIV/HBV co-infection is common, and therefore high infection rates are to be expected in recent immigrants from Africa. High rates of co-infection with HBV and also hepatitis C virus (HCV) are expected amongst HIV positive injecting drug users. For patients with HIV/HCV co-infection, prevention of HBV infection is critical, because this viral infection can be particularly severe and may adversely affect therapy for HIV infection [7].

We therefore carried out a study on all HIV patients attending the blood borne virus clinics and genitourinary clinics in Fife, Scotland to determine the: 1) prevalence of HBV infection in Fife HIV cohort; 2) prevalence of occult HBV infection; 3) immunisation status for HBV in the Fife HIV cohort.

## Materials and Methods

Case notes on all HIV patients attending the blood borne virus and genitourinary clinics in NHS Fife from February 2007 to February 2008 were reviewed retrospectively. NHS Fife serves a population of approximately 280 000. Details on patient demographics, risk category for HIV/HBV acquisition, nadir/current CD4 count, HIV viral load and time since diagnosis of HIV were recorded. HBV serum markers including hepatitis B surface antigen (HBsAg), hepatitis B surface antibody (anti-HBs) and hepatitis B core IgG antibody (anti-HBc), and Hepatitis C markers including hepatitis C antibody and polymerase chain reaction (PCR) for antibody positive patients were tested prospectively if these tests were not carried out in the previous 12 months. Patients with a positive HBsAg result and patients with positive anti-HBc result underwent further testing for HBV DNA by PCR. Alanine transaminase (ALT) levels were also recoded during the time of testing HBV serum markers. ALT levels were considered to be normal if it was less than 40 IU/ml, mildly raised if it was less than twice normal levels or raised if it was more than twice normal levels. HBV vaccination history was obtained from case notes or from direct questioning of patients. Details of anti retroviral medications were obtained from case notes. A total of 70 patients were included in the study; 8 patients were excluded as they had moved away from Fife before all the HBV markers were tested. Continuous variables are presented as mean values and absolute variables are presented as percentages.

## Results

Male to female ratio was 2.3 to 1 (49 males, 21 females). Mean age was 42.6 years (range 20 - 69). Patient ethnicity consisted of 55 (78%) Caucasians, 12 (17%) Africans, and 4 (5%) Asians. Main risk factors for acquiring HIV in this cohort were as follows: 40 patients (57%) acquired HIV by heterosexual contact, 22 (31%) through same sex contact (MSM) and 8 (12%) patients acquired HIV through intravenous drug use. Mean time since diagnosis of HIV was 6 years (1 month - 20 years). Patient variables are shown in Table 1.

**Table 1:** Variables of HIV Cohort in Fife

Characteristic of Variable	Results
Age in years: Mean (range)	42.6 (20 - 46)
Nadir CD4: Mean (range) cells/mm^3^	174 (0 - 673)
CD4 cell count at the time of HBV testing: Mean (range) cells/mm^3^	409 (52 - 1074)
HIV RNA level at the time of HBV testing: Mean (range) copies/ml	29012.0 (< 40 - 1000000)
HCV antibody positivity	9 (12.9%)
HCV RNA positivity	8 (11.2 %)
Current HBV infection/carrier (HBsAg positive, anti-HBc positive)	2 (2.8%)
Past exposure to HBV (HBsAg negative, anti-HBc positive)	16 (22.9%)
Occult HBV infection (HBsAg negative, anti-HBc positive, HBV DNA positive)	2 (2.8%)
Positive history for HBV immunisation	17 (24.2%)
Detectable Anti-HBs in immunised patients	12 (70.6%)
ALT level on HBsAg positive, anti-HBc positive patients: Mean (range) IU/ml	32.6 (10 - 111)
Patients with positive HBV serum markers currently receiving TDF/3TC/FTC regimes	14 (77.8%)

Table 2 breaks down the vaccination figures further: 9 of 22 patients (41%) who acquired HBV through same sex contact (MSM) were vaccinated; 7 patients (78%) had detectable antibody response, with 5 patients (56%) demonstrating sufficient antibody response (> 100 IU/ml) according to BHIVA guidelines; 8 of 48 patients (17%) of other risk groups were vaccinated; 5 patients (62.5%) had a detectable antibody response, with 2 patients (25%) demonstrating an antibody response of more than 100 IU/ml.

**Table 2:** HBV Vaccination Status and Antibody Response in the HIV Cohort in Fife

Category	Vaccination Status	Antibody Response		
		> 100 IU/l	< 100 IU/l	Not tested
**MSM** n = 22 (31%)	Vaccinated: 9 (41%) Not vaccinated: 13 (59%)	5 (56%)	2 (22%)	2 (22%)
**Others** n = 48 (69%)	Vaccinated: 8 (17%) Not vaccinated: 40 (83%)	2 (25%)	3 (37.5)	3 (37.5%)

## Discussion

Hepatitis B is one of the most common human pathogens worldwide. Although hepatitis B is a double-stranded DNA virus, it has several common features with HIV. After entering the hepatocyte, viral DNA is integrated into the host genome. Viral RNA is translated by HBV reverse polymerase into viral DNA and transcribed into viral proteins. Reverse transcription may be inhibited by nucleotides reverse transcriptase inhibitors. Integration of the virus into the host genome of hepatocytes and CD4+ T-cells prevents its eradication. Finally, the mechanisms for development of resistance are very similar for both viruses.

There is considerable variation of HBV/HIV co-infection among geographical regions and risk groups. Our study showed that 25.7% of HIV patients had evidence of exposure to HBV (anti-HBc positive) with 2.8% currently co-infected (HBsAg positive, anti-HBc positive) and 22.9% showing evidence of past exposure (HBsAg negative, anti-HBc positive). The combination of HBsAg negativity and anti-HBc positivity is traditionally interpreted as indication of prior hepatitis B infection with clearance. More recently, it has become apparent that HBV DNA can be detected in the serum from at least some individuals who exhibit this combination [8]. The term occult infection is used to describe this picture. Among our patients, 2.8 % had a detectable HBV DNA and anti-HBc despite a negative HBsAg, thus fulfilling the criteria for an occult infection.

Studies indicate that the natural history of HBV is altered in patients with HIV disease due to immune dysfunction which increases the risk of becoming a chronic HBV carrier [9]. During HIV infection, an isolated anti-HBc can be validly interpreted as immunity with past infection, false positive core antibody, late phase of an acute HBV infection when HBsAg has disappeared or chronic HBV infection with an undetectable HBsAg or a low level of HBsAg titre. However, it is unlikely for the anti-HBc to be falsely positive in high risk persons [10]. Furthermore, in a recent long term follow-up study of anti-HBc/HIV positive patients of whom transient HBsAg positivity developed in 24%, HBV DNA became positive in 60% and about a third of these patients had active liver disease, thus suggesting that an isolated anti-HBc most likely represents chronic infection [11]. It is therefore vital to test for anti-HBc in patients with negative HBsAg in HIV disease, partly to determine which patients would be likely to develop occult infection or reactivation of HBV. Also, HBV markers including HBV DNA (in anti-HBc positive patients) should be rechecked annually, especially if the liver function tests become abnormal.

In patients requiring HIV therapy who are HBsAg positive, therapy would normally include tenofovir (TDF), or a combination of tenofovir with either lamivudine (3TC) or emtricitabine (FTC) as part of, or in addition to, their antiretroviral regimen [5]. Withdrawal of lamivudine may result in an acute exacerbation of hepatitis that may be sufficient to precipitate liver decompensation [12,13]. There are also reports of clinical reactivation of HBV infection in HIV infected patients with isolated anti-HBc as the only serological marker after lamivudine withdrawal [14]. This study showed that 77.8% of HIV patients in Fife who had a positive anti-HBc result were on TDF, TDF/FTC or 3TC containing regimens. In this group, all patients with a positive HBsAg or HBV DNA in addition to anti-HBc indicating current infection or occult infection were on appropriate anti-retroviral therapy. Rest of the patients with a positive anti-HBc, but not on any anti-retroviral therapy had good CD4 count results (> 500 cells/mm^3^). BHIVA guidelines recommend that patients who are HBV/HIV co-infected, with a CD4 count less than 500 cells/mm3 should commence HAART. Only exception to this may be the patient with a CD4 count 350 - 500 cells/mm^3^, HBV DNA less than 2000 IU/ml with normal ALT levels and no evidence of fibrosis or hepatic inflammation. In this situation, close monitoring is essential [5].

HBV is immunopathic (the immune response to the virus causes most of the liver damage) and therefore in HIV infection there is often a decrease in the serum transaminases and a decrease in the histological inflammatory indices. Mean ALT level amongst our patients with a positive HBsAg or anti-HBc was 32.6 IU/ml which is within the normal range. It is therefore important to test for HBV in HIV disease even if the liver enzymes are not elevated. However, co-infection with HIV is usually accompanied by an increase in HBV replication [15,16], and at very high levels of viral replication the virus may have a direct cytopathic effect.

In HIV infected patients, chronic HBV has an unfavourable course compared with those who do not, and the risk of liver-associated mortality is significantly increased. In addition to increased mortality, HIV co-infection accelerates the progression of hepatitis B and increases the risk of cirrhosis. Therefore, prevention is vital and HBV infection is preventable by vaccination. HIV positive patients however respond less well to vaccine, especially when the CD4 count is low. Also, protective antibodies are lost more quickly [17]. Some authors therefore recommend a conventional vaccination dose for patients with CD4 counts of more than 500 cells/mm3 (20g at months 0, 1 and 12), and an intensive schedule for patients with CD4 counts of less than 500 cells/mm3 (20g at months 0, 1, 2 and the last dose between months 6 and 12) [17]. Double dose vaccine may also improve response rates. However, if the CD4 cell count is less than 200 cells/mm3, the patients should receive antiretroviral therapy first and immunisation thereafter [5]. Our study showed that 24.2% of patients were vaccinated for HBV. Table 2 shows the breakdown figures clearly. Recently published Quality improvement Scotland (QIS) standards recommend that at least 70% of all MSM who are attending sexual health services and not known to be immune to HBV should receive at least one dose of HBV vaccine [18]. Only 41% of our MSM were vaccinated and only 56% of these patients had a sufficient antibody response of more than 100 IU/ml (BHIVA standards). Vaccination figures fall well short for other risk groups with only 17% being vaccinated with 25% showing a sufficient antibody response. Therefore, improvements could be made in the field of prevention with vaccination and monitoring the immune response in our patients.

In conclusion, the prevalence of HBV infection in the Fife HIV cohort is 5.6% of which 2.8% of patients had evidence of occult infection. Furthermore, 22.9% of patients had evidence of previous exposure to HBV and were at risk of reactivation. Although HBV is preventable by vaccination, only a minority (24.2%) of patients had been vaccinated against it.

We therefore recommend adopting the following steps from recently updated BHIVA guidelines [5] on co-infection with HIV and HBV infection during assessment and management of all HIV positive patients: 1) all patients should have serum markers for HBV (HBsAg, anti-HBc and/or anti-HBs) checked within a month of diagnosis and should have further tests if the result is positive; 2) all those negative ones for HBV markers should be immunised and anti-HBs checked at 6 - 8 weeks post vaccination (ART naive patients with a CD4 count of less than 200 cells/mm3 should receive combination therapy first and with immune recovery, immunisation subsequently); 3) following successful immunisation, anti-HBs levels should be checked yearly and booster doses of vaccine should be given to those with anti-HBs level of less than 100 IU/l; 4) persons who fail to seroconvert to HBV vaccine and are at continuing risk of HBV infection should have annual HBV markers performed ( HBsAg and anti-HBc); 5) all patients who are anti-HBc positive but HBsAg negative are at risk of HBV reactivation, thus HBV markers including HBV DNA should be rechecked annually or if the liver function tests become abnormal; 6) decisions on appropriate ART combinations should be made after the results of HBV markers.
